# Recurrent Craniocervical Pseudogout: Indications for Surgical Resection, Surveillance Imaging, and Craniocervical Fixation

**DOI:** 10.7759/cureus.511

**Published:** 2016-02-24

**Authors:** Amitoz Manhas, Prashant Kelkar, Joseph Keen, Steven Rostad, Johnny B Delashaw

**Affiliations:** 1 Neurosurgery, Swedish Neuroscience Institute; 2 Pathology, CellNetix; 3 Chief of Neurosurgery, Swedish Neuroscience Institute

**Keywords:** pseudogout, calcium pyrophosphate dehydrate, occipitocervical

## Abstract

Background: Calcium pyrophosphate dihydrate (CPPD) crystallization is known to occur in the spine, leading to the development of visible calcification as seen by imaging. Occasionally, the deposition of this material can lead to larger accumulations that are seen as masses in the articular processes, intervertebral discs, and posterior longitudinal ligaments. A particularly significant manifestation of this process is at the craniocervical junction, where symptomatic presentations can arise.

Clinical presentation: A 74-year-old woman presented after several falls from standing, complaining of leg and arm weakness. Imaging revealed a mass arising from the C1-C2 articulation dorsal to the dens, extending to the clivus. The mass compressed the medulla and cervicomedullary junction.

Intervention: The patient underwent a left, far lateral craniotomy with C1 laminectomy to approach the cervicomedullary junction. The mass was cyst-like and contained scattered crystals and amorphous material consistent with pseudogout. There were no cells with an elevated Ki-67 index. The patient’s symptoms and exam improved at follow-up two months later. However, seven months after surgery, she declined once again and was found to have a recurrence.

Conclusion: A subtotal resection of pseudogout may lead to recurrence. The recurrence can occur in a rapid fashion. Serial MRIs are indicated following resection. Occipitocervical fusion could reduce the likelihood of recurrence in such cases.

## Introduction

The deposition of calcium pyrophosphate dihydrate (CPPD) crystals in the articular processes, intervertebral discs, posterior longitudinal ligament [1-2], and ligamentum flavum of the spine have been reported in the literature since the 1970s when the condition was described as articular chondrocalcinosis[3] with etiologies that ranged from hereditary hemochromatosis, hyperparathyroidism, diabetes mellitus, and gout. Case reports have shown that some nodular opacities by x-ray imaging of the spine have been removed and were found to have included tophaceous material, displaying positive birefringence under polarized light microscopy. The prevalence of CPPD is thought to increase with age, is associated with osteoarthritis without osteoarthritis progression, and is believed to be associated with hyperparathyroidism, hemochromatosis, hypomagnesemia, and hypophosphatasia [4].

Clinical manifestations of this disease have been primarily due to compression of the spinal cord and nerve roots, causing myelopathy or radiculopathy. More commonly, presenting patients are not symptomatic, but instead have incidental radiographic findings. Chang, et al. examined 513 consecutive patients and found an overall prevalence of 12.5%, increasing with age (p<0.0001), to a prevalence of 49% in those older than 80 years [5]. Another is a case-control series comparing patients with known peripheral joint CPPD to those without, which found that neck pain was significantly more common in subjects with known peripheral CPPD, with periodontoid calcification being six times more common [6]. The largest case series of craniocervical junction pathology reported was by Fenoy, et al., where 21 patients were followed for an average of 17.5 months. Nearly all underwent transoral-transpharyngeal resection of the anterior arch of C1, the odontoid process, and the mass lesions. The authors cited gross-total resection in all of the patients in their series with no residual mass on follow-up imaging [7].

## Case presentation

### History

A 74-year-old woman presented having had several falls, complaining of right leg and arm weakness, and was found to have a 17 x 17 x 23 mm mass arising from the C1-C2 articulation dorsal to the dens, extending up to the clivus. Imaging demonstrated marked compression and posterior displacement of the medulla and cervicomedullary junction. The patient did not present with a history of arthralgias, malignancy, or urinary or bowel complaints. She formerly smoked (history of 0.5 packs/day for 20 years . She stopped smoking seven years prior to presentation to the hospital in February 2014) and had previously had an anterior cervical discectomy and fusion of C3/4, C4/5, C5/6, as well as shoulder and knee surgeries for osteoarthritis. She did not have other relevant past personal or family medical history.

### Examination

The patient had diminished sensation to light touch and proprioception on the right side, with decreased strength in the right upper extremity (4-/5) and right lower extremity (4/5). She also was hyperreflexic on her right side.

**Figure 1:**
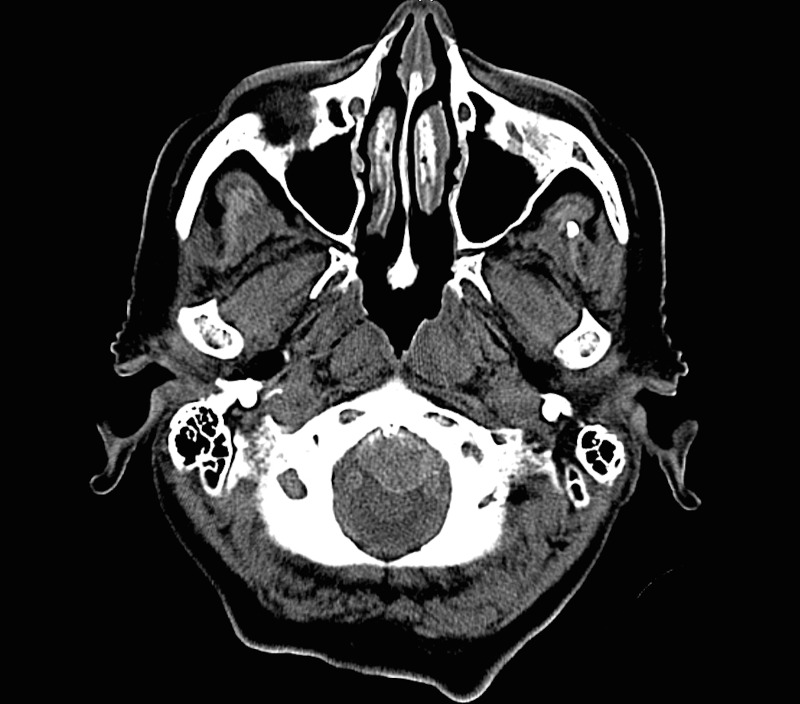
February Axial CT Image 1 Foramen magnum

**Figure 2:**
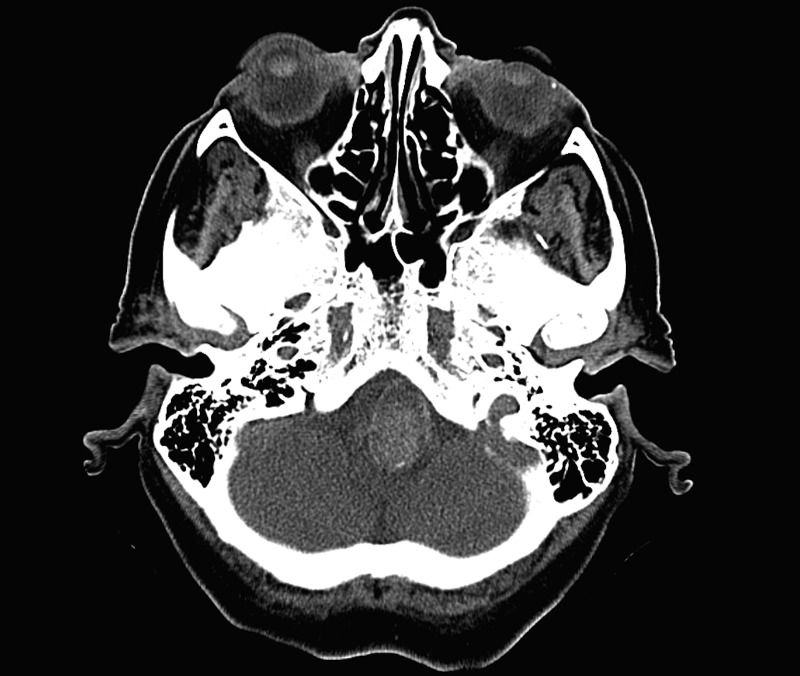
February Axial CT Image 2 Posterior fossa

**Figure 3:**
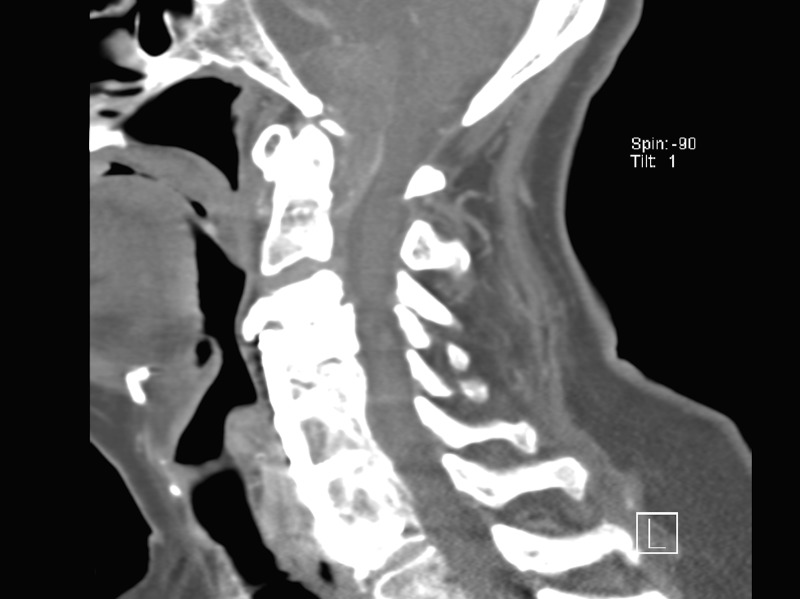
February Sagittal CT Image Cervicomedullary junction

A CTA was completed to determine if there was an aneurysm arising at the level of the abnormality. There was no discernable filling of the mass by CTA. There was no conspicuous, contiguous bone erosion or destruction in the skull base next to the mass. A well-circumscribed, peripherally hyperdense, extra-axial lesion measured approximately 16 x 21 mm at its largest dimension. Additionally, there was an air-fluid level within the mass, indicating that a portion of it was cystic. Figures 1-3 are representative images of the pathology on the initial CT imaging.

**Figure 4:**
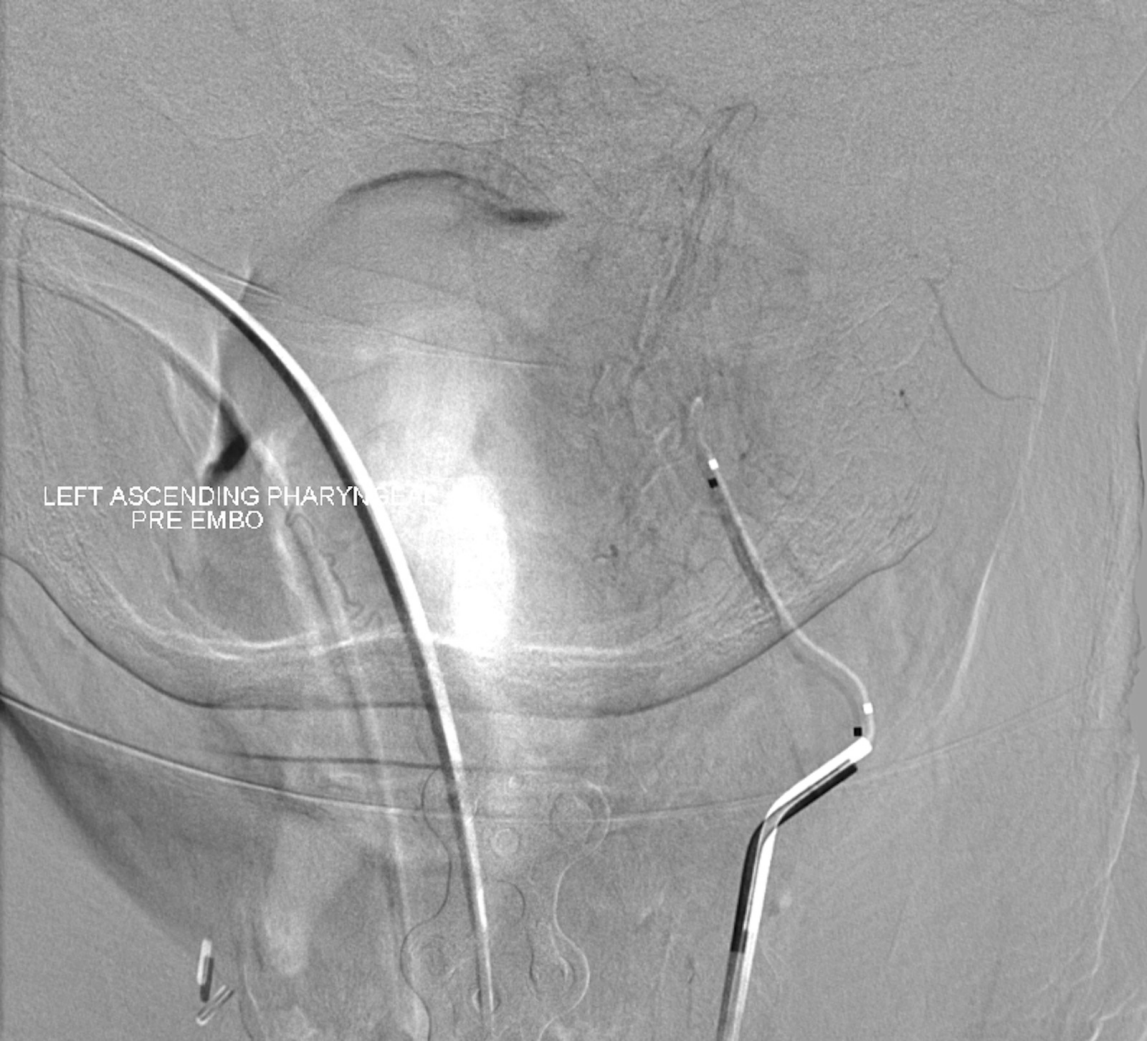
Ascending pharyngeal artery embolization

For surgical planning, an angiogram was completed. A distinct blush through the ascending pharyngeal artery was found by angiogram, with partial filling of the body of the mass. The vessel was subsequently embolized completely. A left, vertebral artery catheterization can be seen in Figure 4, with a Synchro 2 wire in the ascending pharyngeal artery, demonstrating filling of the tumor. There was stagnation of flow into the neovascularization consistent with neoplasm. Embolization was completed with 300-500 micro embospheres (approximately 4 cc total) through a microcatheter, with no further filling of the ascending pharyngeal artery.

**Figure 5:**
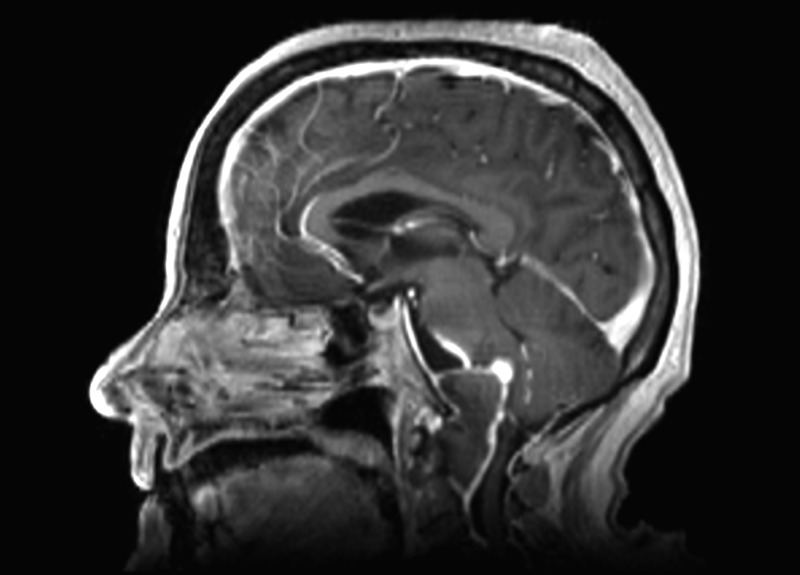
February Sagittal MRI Craniocervical junction mass

**Figure 6:**
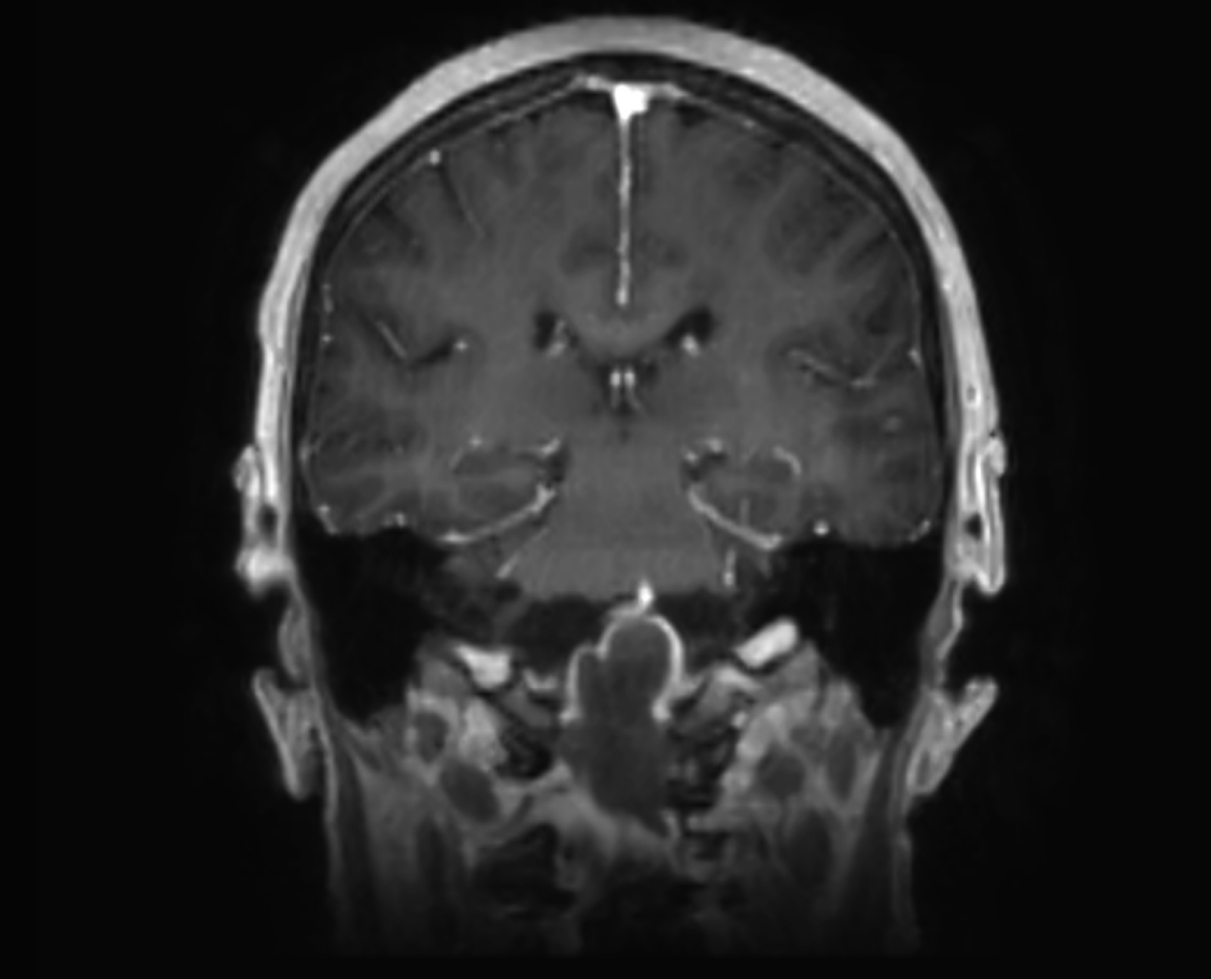
February Coronal MRI Craniocervical junction mass

By MRI, the contents of the mass were isointense on T1 and hypointense on T2, with thin peripheral enhancement, as seen in Figures 5-6. The mass extended from the lower aspect of the clivus caudally to the lower aspect of C2. This measured 4.2 cm craniocaudally, 2.7 cm anteroposteriorly, and 3.1 cm transversely. There were severe compression and posterior displacement of the cervicomedullary junction, to a diameter of less than 6 mm of the central canal. The vertebral arteries could also be seen splayed laterally. There was a nodular focus of enhancement noted at the top of the mass near the upper medulla. On axial T2 FLAIR sequence images, edema was seen within the brainstem. The 4th ventricle was flattened, but there was no ventriculomegaly.

### Operation

The patient underwent a left, far-lateral craniotomy with C1 laminectomy to approach the cervicomedullary junction. The patient was placed in a three-quarters prone position. Facial nerve monitoring was initiated prior to incision. An incision was made above the pinna, lengthened to behind the asterion downward in a paramedian plane toward C2 and then forward inferior to and in front of the mastoid tip. The craniectomy was done using a high-speed drill. The C1 lamina was removed laterally to the foramen transversarium. Once the condyle was visualized, it was drilled, taking care to recognize the entry of the vertebral artery into the dura, and the sigmoid sinus and dura exposed posterior to these structures. The dura was opened at the foramen magnum, the cisterna magnum was opened, and then the dural opening was extended along the axial plane of C1 and superiorly along the cerebellar hemisphere, posterior to the sigmoid sinus. The cerebellar hemisphere at the level of the foramen magnum was lifted superiorly and posteriorly. Microdissection under the microscope allowed the brainstem to be visualized as well as the vertebral artery, distorted anteriorly by the visible mass. Cranial nerves IX, X, XI, and XII were visualized stretched laterally by the mass. Examining the mass along its limits, it appeared to be extradural. The mass was opened using an #11 blade and was noted to have a firm texture, gray and yellow in appearance. Specimens were sent to pathology, and the preliminary diagnosis was an avascular lesion, consistent with pseudogout. The tumor was debulked until the brainstem was no longer compressed. The limit of the mass was actually the dura, and therefore, the mass continued to be debulked in the extradural plane. The brainstem surface was somewhat adherent to the surface of the dural plane, and, therefore, it was not separated from this surface. The mass was removed using various curettes, suction, and microscopic instruments. A portion of the mass wall was also sent for histological evaluation.

### Pathological examination

**Figure 7:**
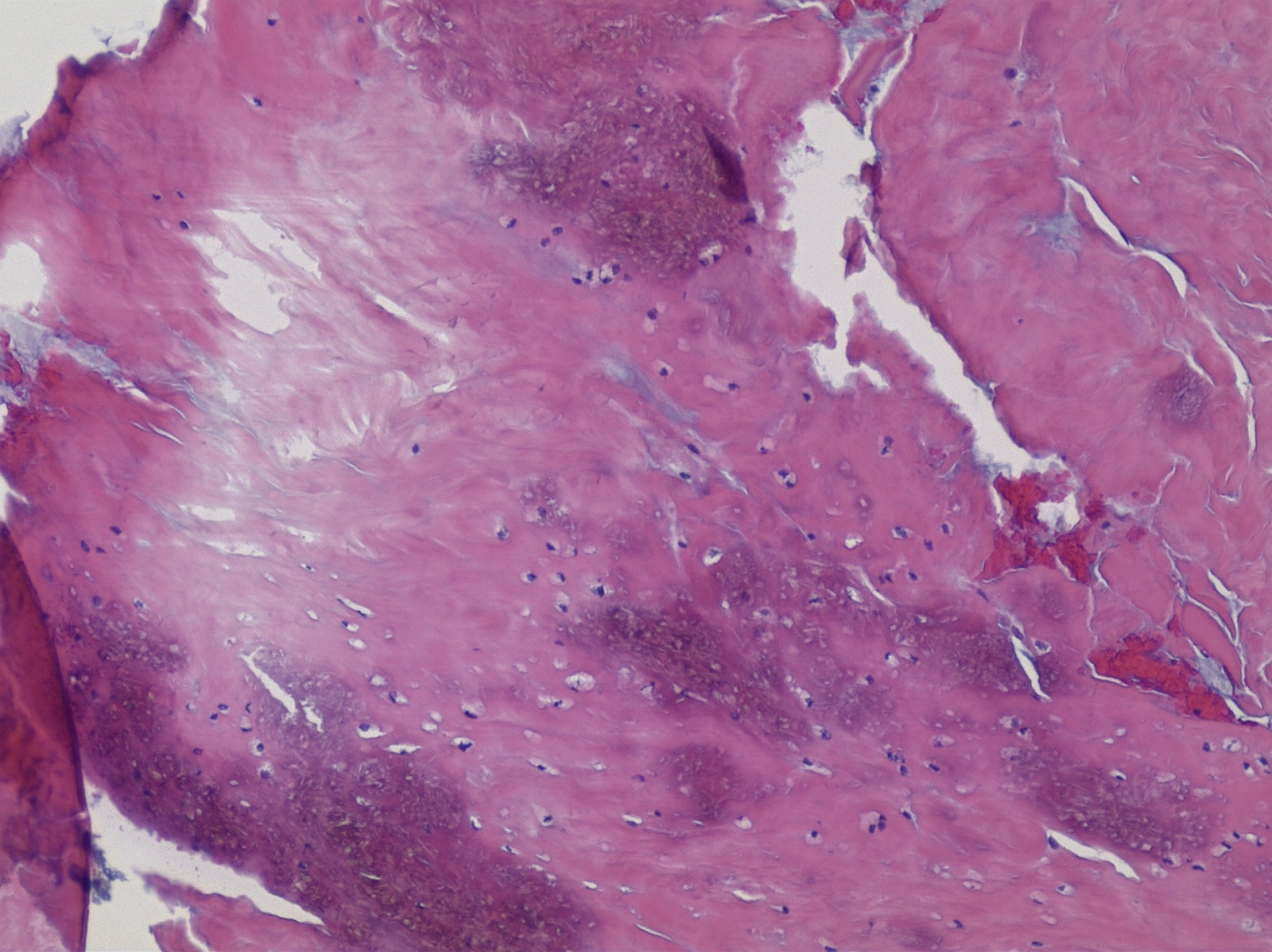
Pathology Slide - Synovial Chondroid Tissue

**Figure 8:**
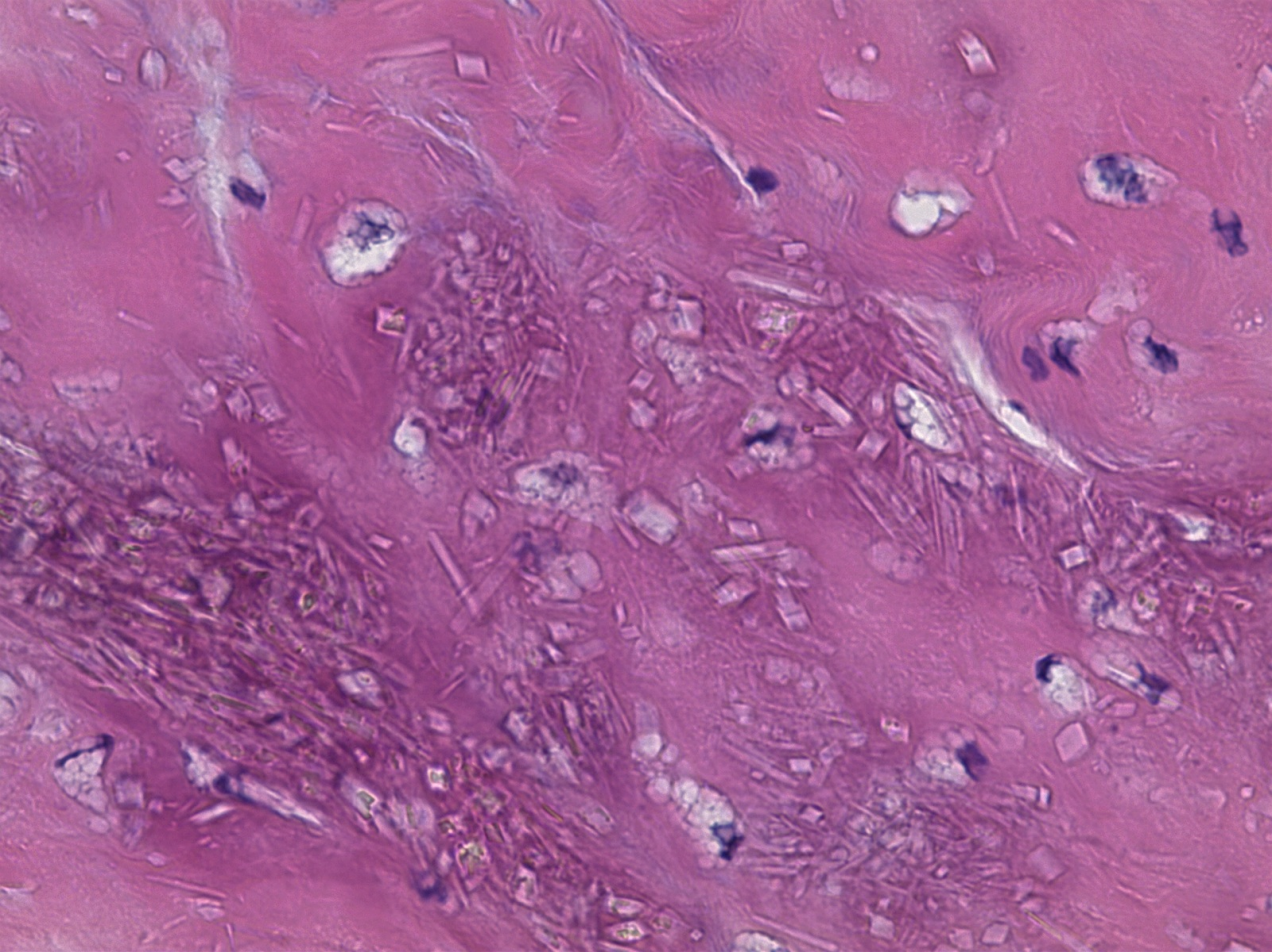
Pathology Slide - Birefringent Calcium Pyrophosphate High power showing crystals within synovial-chondroid material. The crystals are birefringent on polarization optics, consistent with calcium pyrophosphate.

**Figure 9:**
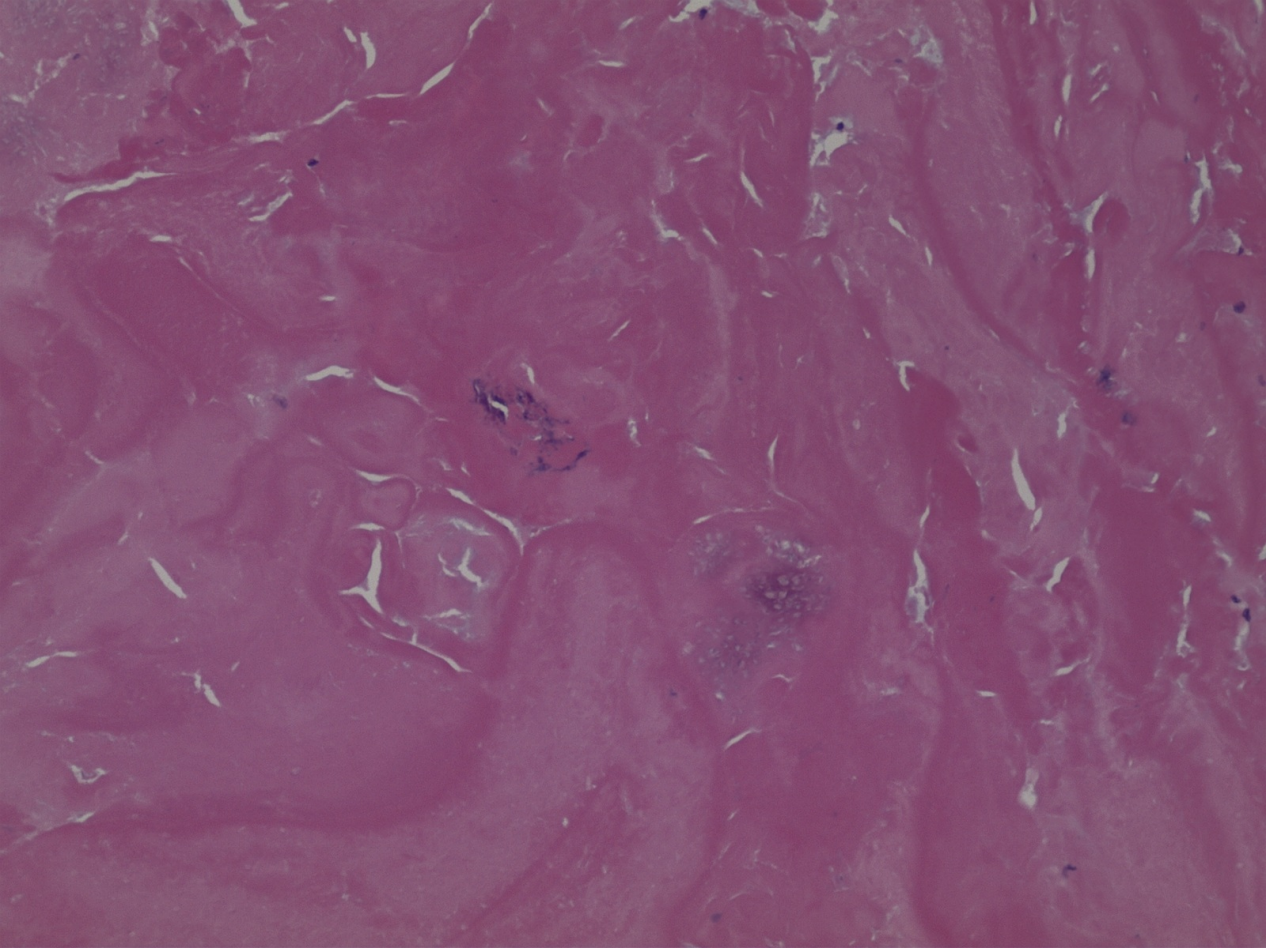
Pathology Slide - Amorphous material Amorphous material within acellular region of the  tissue (no crystals visible)

**Figure 10:**
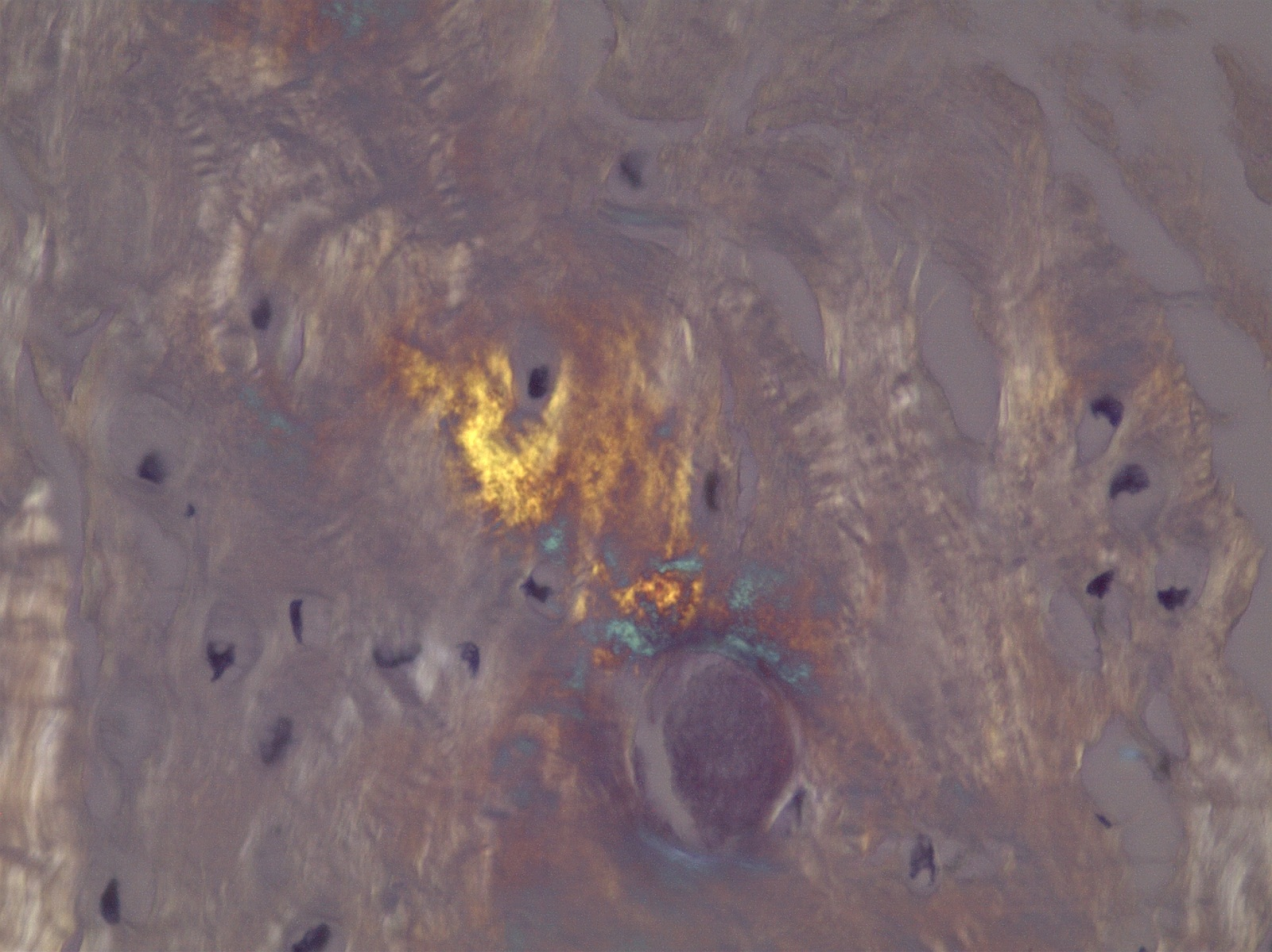
Pathology Slide - Congo Red Stain Congo red stain of 3, with polarization, showing characteristic “apple green-orange” staining of amyloid.

The material within the cyst was composed of scattered basophilic crystals and amorphous material consistent with pseudogout (Figure 7). The specimen included synovium with extensive fibrinoid and some cartilaginous degeneration. A Congo red stain demonstrated scattered amyloid deposits with polarization optics. It did not display positive Pankeratin staining, which is seen in cases of chordoma. The Ki-67 labeling index was low (approximately zero). Additional pathological examination confirmed the diagnosis of pseudogout, as shown in Figures 8-10.

### Postoperative course

**Figure 11:**
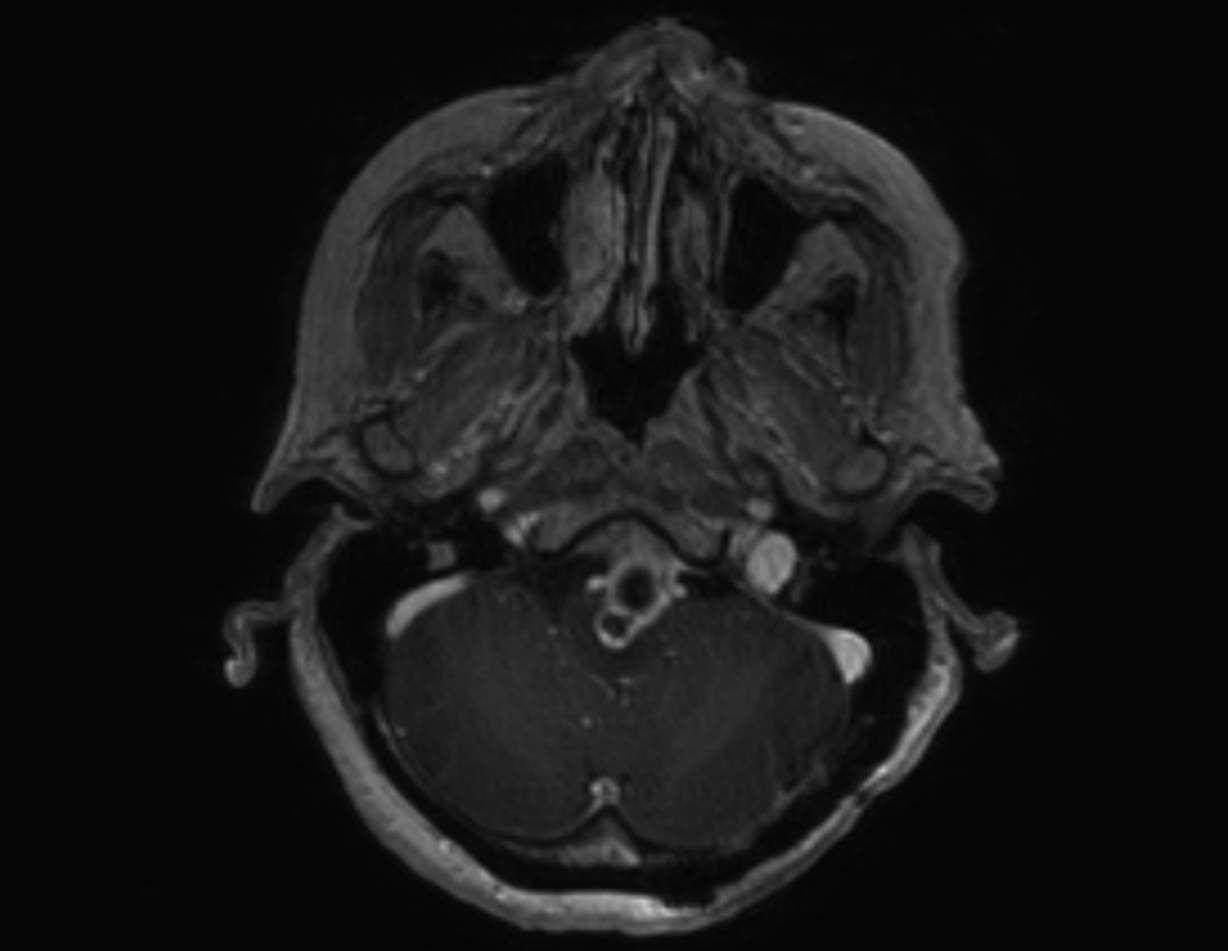
September Axial MRI Image 1 Posterior fossa

**Figure 12:**
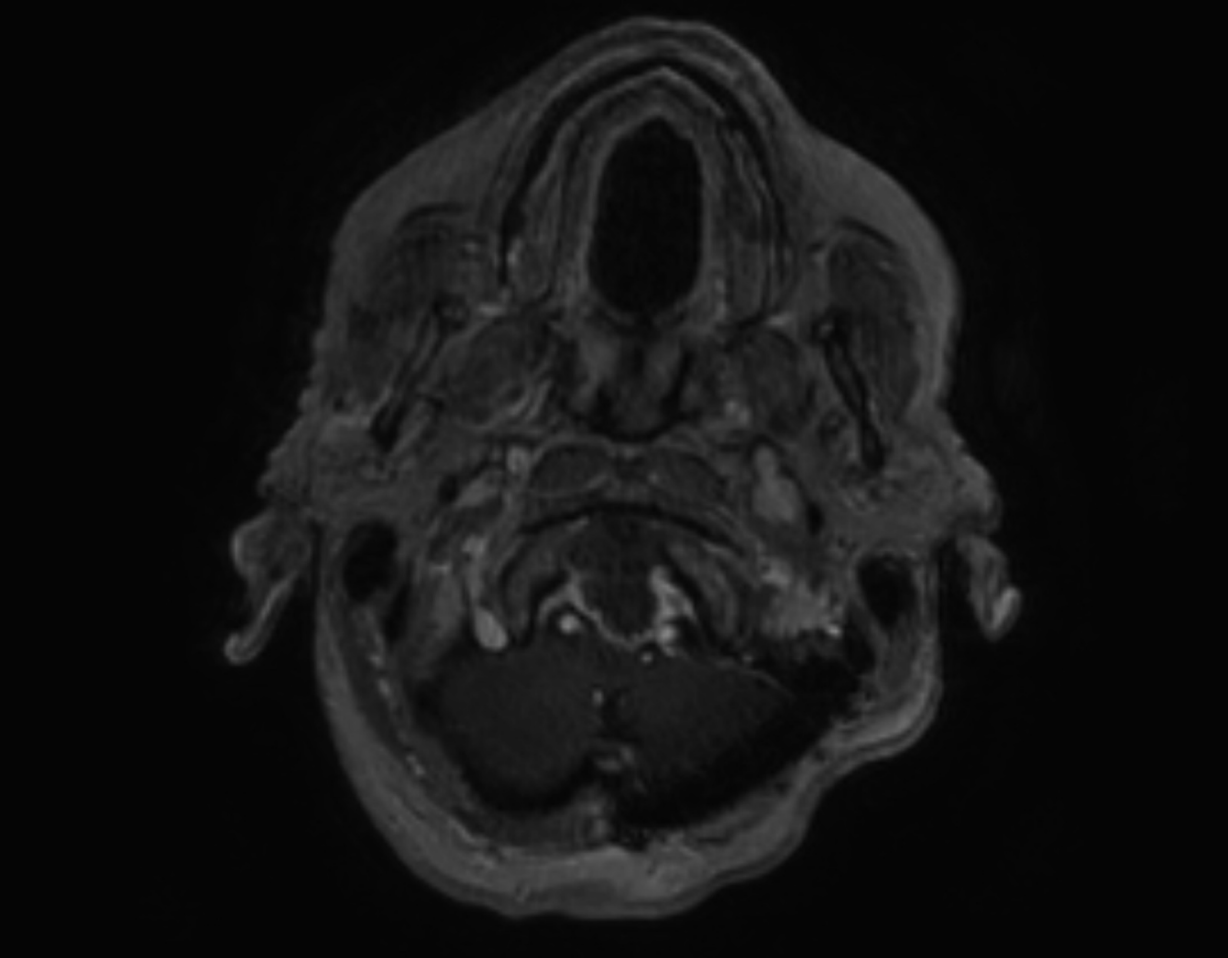
September Axial MRI Image 2 Foramen Magnum

**Figure 13:**
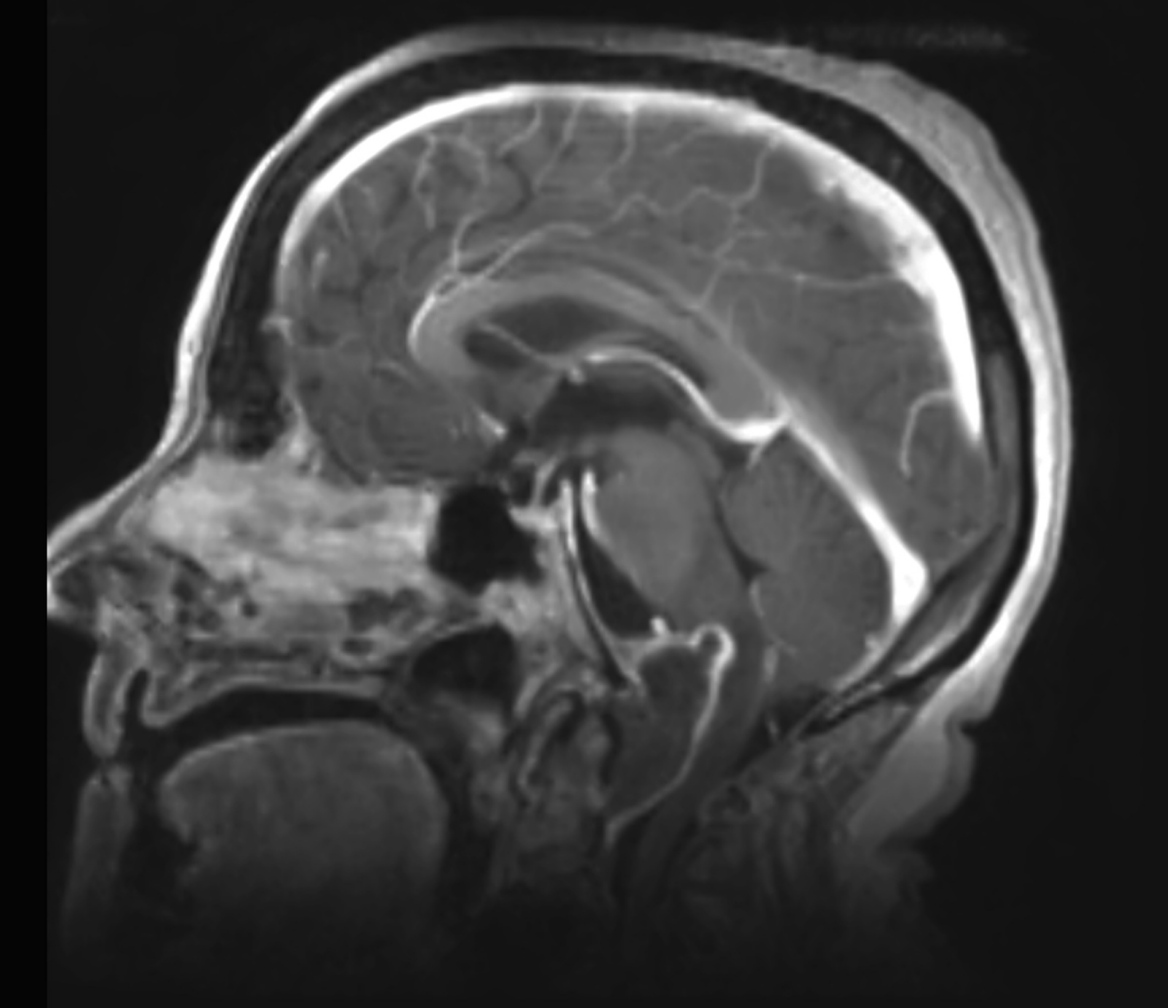
September Sagittal MRI Enhancing mass

**Figure 14:**
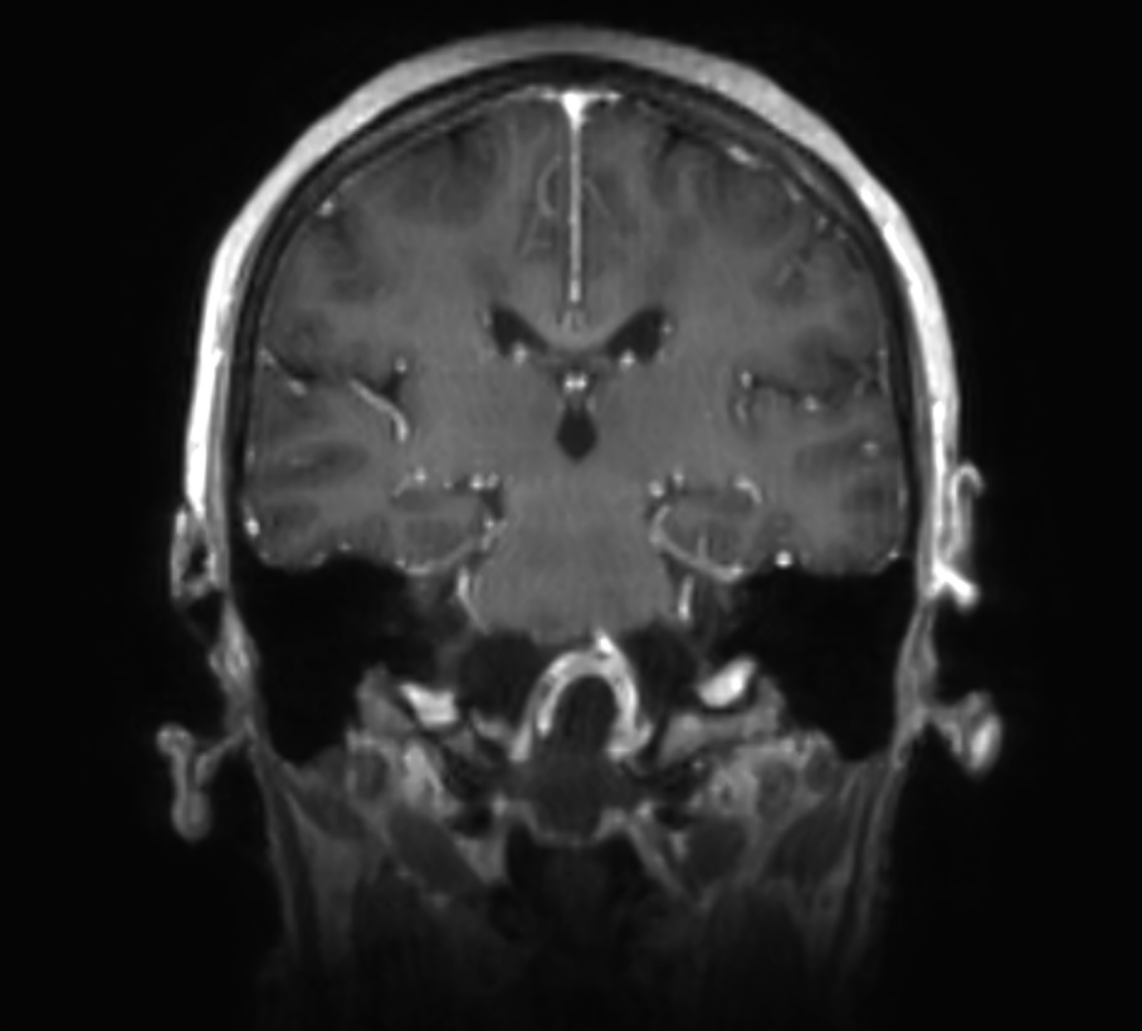
September Coronal MRI Enhancing mass

The patient improved following surgery and was able to ambulate with a walker when she was seen in clinic two months later. She presented back seven months after surgery in September with a five-day history of progressive confusion, diplopia, and worsening weakness in her right arm and leg. A repeat MRI was completed which showed the lesion had grown to 2.8 x 4.9 x 2.1 cm in size, causing more compression of the medulla and upper cervical spinal cord. There was no evidence of ventriculomegaly. A complex cystic and solid enhancing mass was again seen on the repeat imaging at that time. The lesion appeared to derive from the C1-C2 articulation. There was marked peripheral enhancement. There was also a mass effect on the medulla and craniocervical junction. Extensive, abnormal T2 signal was present within the medulla and pontomedullary junction, which had worsened since the February prior study (Figures 11-14). 

**Figure 15:**
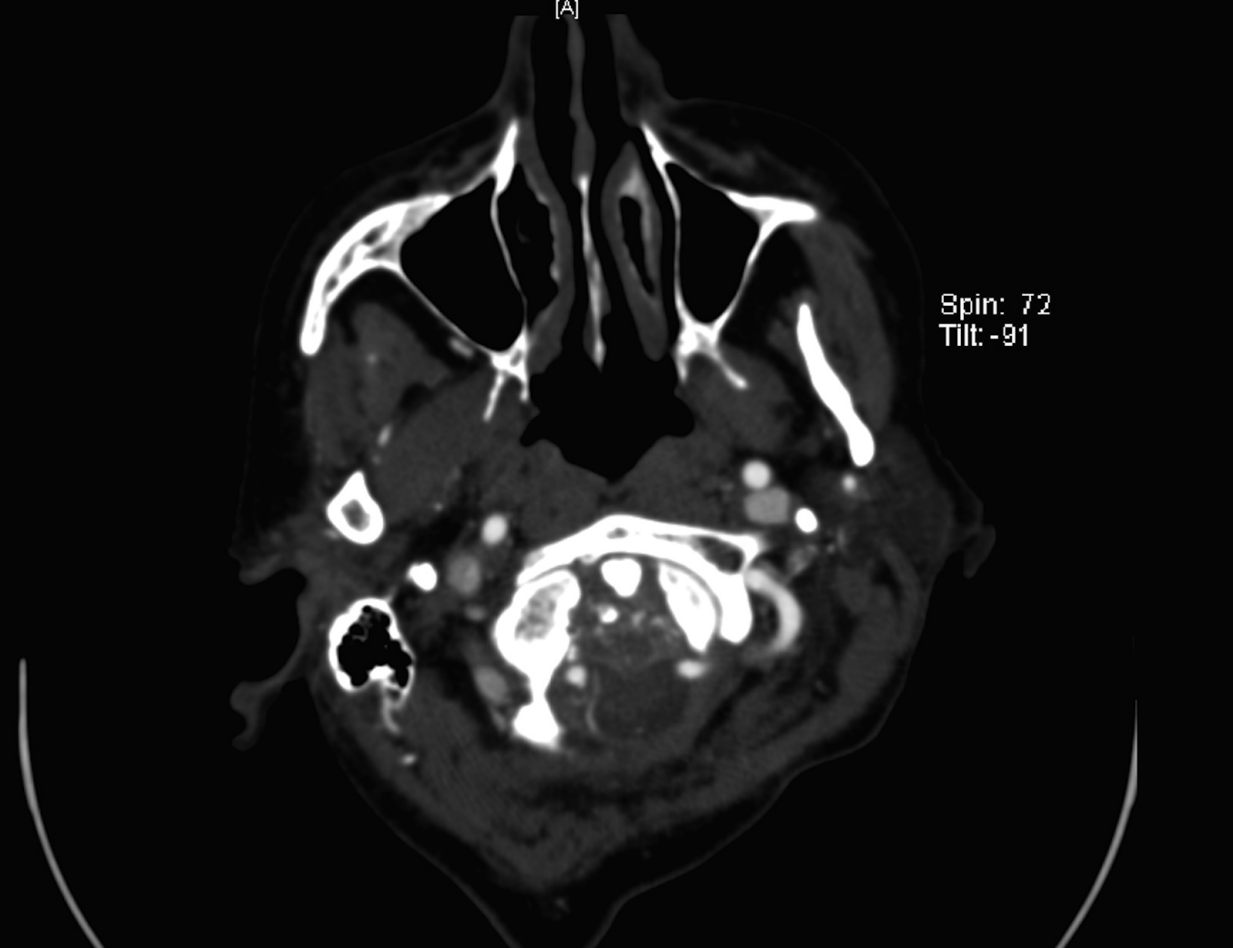
September Axial CT Retrodental mass

The cervical spine bony anatomy was imaged using a CTA, which revealed essentially stable cervical spine bony anatomy and alignment. The vertebral arteries appeared externally compressed, but there was no change in the appearance of the cervical vertebral segments. A large, partially calcified, retrodental mass could be seen extending cranially into the intracranial space along the posterior margin of the lower clivus as seen in the MRI. There was no significant pathology identified involving the intracranial vertebrobasilar circulation (Figure 15).

The patient underwent surgery to remove the mass and cyst wall through the previous far lateral approach and then had a posterior fixation from the occiput to the 6^th^ cervical segment, thus providing occipitocervical stability. At the time of tumor resection, the mass appeared to be more fibrous and adherent as compared to the prior surgical resection, with multiple layers of tissue within the mass. It was debulked and could not be completely resected given its adherence to all surrounding structures, particularly the brainstem. SSEP monitoring and facial nerve monitoring were used during surgery. The pathological results were identical to the previous report earlier in the year. Her course in the hospital following the surgery was uncomplicated. She continued to improve with regard to her strength, and follow-up imaging at one year demonstrated no recurrence at the site of surgery.  

## Discussion

Kawano, et al. postulated that CPPD may arise from the deposition of metaplastic chondrocytes in locations such as the ligamentum flavum [2]. A caveat noted by previous authors [3] with regard to the preparation of this tissue for pathological examination is that decalcification processing will dissolve CPPD crystals, making diagnosis difficult to make. Therefore, the typical permanent method of treating bony or fibrocartilaginous tissue sent to pathology may make a diagnosis indeterminable in cases where a small amount of tissue is given to pathology for analysis. Currently, there are no effective treatment modalities of systemic CPPD, except for management of joint pain. There are no medicines to reduce the burden of crystals in joints that lead to inflammation and degeneration [8].

The treatment of CPPD disease at the craniocervical junction has been an infrequent occurrence. The most common types of pathology found at this level of the neuroaxis are those relating to inflammation such as rheumatoid arthritis, Paget’s disease, degeneration as in ankylosing spondylitis, diffuse idiopathic skeletal hyperostosis (DISH) [9], basilar invagination, congenital abnormalities as in hypoplasia, platybasia, narrowing of the foramen magnum secondary to achondroplasia or Chiari malformations, or malignant etiologies including meningioma, clival chordoma, metastatic disease, schwannoma, and chondrosarcoma.

Chang, et al. reviewed the average retro-odontoid soft tissue thickness in their cohort and found it to be 2.4 mm, with calcified, hyperdense areas presumed to be CPPD beginning at an average of 3.4 mm. Therefore, the question becomes, which hyperdense, calcified region is likely to enlarge or become a CPPD nodule? Those subjects included in the Chang, et al. prospective study were those involved in trauma, and there were no histopathological correlates to the imaging findings [5]. Phosphocitrate has been studied and found to reduce pseudogout formation in animals, but no such study has been performed in humans. It is uncertain if this study is helpful for our patient report, but is available in the literature [10].

Zunkeler, et al. in 1996 published a review of cases on CPPD at the craniocervical junction, at that time stating that there had only been four such cases [3]. The three known cases that had reported surgical approaches were all through a transoral, transpharyngeal method. A total resection was completed using a high-speed drill through the odontoid with opening of the tectorial membrane to remove the portions of the tumor extending further. No dural entry was noted to have been made. The largest series of patients reported were 21 over the course of 30 years at another institution [7]. Other case reports of this diagnosis exist in the literature, and many of these patients were neurologically asymptomatic at the time of their presentation. Elderly women are more likely to have this finding. The likelihood of recurrence has not been explored. 

## Conclusions

The patient in this case review had a subtotal resection, and recurrence was seen in less than one year. It did not exhibit a high proliferative index by histological examination despite the recurrence. Following resection less than a year later, followed by surgical fixation, the patient has not had a recurrence more than one year later and is otherwise neurologically improved. The patient is continuing to be followed with serial MRI studies. Questions that remain to be answered include: How often must thickened retro-odontoid soft tissues be followed? Is occipital-cervical fusion necessary with initial resection to prevent C1-C2 or occipitocervical instability? And finally, is a gross-total resection of the capsule needed to avoid recurrence of this pathology?
